# Using Q-Methodology to Identify Core Illness Experience Dimensions in Treated Lymphoma Patients

**DOI:** 10.3390/curroncol33080442

**Published:** 2026-07-23

**Authors:** Shanshan Yu, Rongxue Ma, Yanjin Liu, Yuting Chen, Xinyu Lu, Yuxi Zhang

**Affiliations:** 1 School of Nursing, Nanjing University of Chinese Medicine (NJUCM), Nanjing 210023, China; 20231017@njucm.edu.cn (S.Y.);; 2 Geriatric Respiratory Ward, the First Affiliated Hospital of Nanjing Medical University (Jiangsu Province Hospital), Nanjing 210029, China

**Keywords:** lymphoma, experience, experience-based co-design, Q-methodology, patient-reported outcome measures

## Abstract

Lymphoma currently ranks first in incidence among haematological malignancies, yet few have revealed the core dimensions of the illness experience which patient-reported during treatment. This study aimed to identify those core dimensions using Q-methodology, which combines qualitative and quantitative approaches. A concourse of 59 statements was developed through literature review and dual-perspective interviews with clinicians and patients, then refined to a 39-item Q-set. Seventeen hospitalized lymphoma patients performed Q-sorting, and data were analyzed by principal component analysis with varimax rotation. Four distinct factors emerged: need for a healing environment amid severe adverse reactions; active adaptation and positive coping with treatment; chronic symptom distress and fear of recurrence; and resilient persistence under heavy financial pressure. These findings reveal complex and diverse subjective experiences of lymphoma patients. Differentiated management approaches should be applied to patients with distinct experience patterns, such as improving the physical hospital environment, fostering active coping and post-traumatic growth, alleviating recurrence anxiety, and mitigating financial toxicity.

## Simple Summary

Lymphoma is the most common blood cancer, yet little is known about how patients truly feel during treatment. In this study, we used a special method that lets patients rank their personal experiences. We found four distinct patterns: some patients care deeply about the hospital environment amid strong treatment side effects; some adapt actively and cope positively with treatment; others are troubled by chronic symptoms and fear of recurrence; and some bear heavy financial pressure while persisting resiliently. These findings help nurses and doctors provide more personalised support, improve hospital care, and develop better tools to measure what matters most to patients.

## 1. Introduction

Global estimates indicate that over the past 30 years, the incidence and mortality of hematologic malignancies have increased significantly and pose substantial threats to people’s lives [1]. Lymphoma is a common malignant tumor of the hematopoietic system, encompassing Hodgkin lymphoma (HL) and non-Hodgkin lymphoma (NHL). According to global cancer burden data released by the International Agency for Research on Cancer (IARC) in 2022, NHL incidence surpassed leukemia to rank tenth, while its mortality rate ranked eleventh [2]. In 68.85% of the 185 countries studied, NHL represents the hematologic malignancy with the highest lifetime risk of incidence (LRI) [3]. The incidence and mortality rates of HL are relatively low compared to other hematologic malignancies; however, the Global Burden of Diseases, Injuries, and Risk Factors (GBD) Study 2019 showed that China accounted for 10.8% of new cases and 9.8% of deaths due to HL [4].

The patient journey encompasses the entire process from symptom onset through diagnosis, treatment, and follow-up. For lymphoma patients, this journey is characterized by highly diverse symptom presentations and a marked decline in health-related quality of life (HRQOL), owing to the disease’s widespread involvement of multiple sites, complex pathological classification, and adverse treatment reactions. Although numerous studies have investigated lymphoma patients using quantitative or qualitative methodologies, the vast majority remain confined to aspects such as symptoms, health-related quality of life, and unmet needs [5,6,7]. Scarcely any research has focused on comprehensively examining patient-reported outcomes concerning the illness experience of lymphoma patients during the active treatment phase. As patients receiving active treatment maintain regular contact with healthcare providers and receive timely clinical management, in-depth exploration of their subjective experiences during this period is critical for optimizing care delivery [8].

Q methodology, first developed by William Stephenson in 1935, represents a systematic approach to quantifying subjective viewpoints by inverting the conventional factor analytic paradigm: rather than correlating test variables across large samples of individuals, it correlates individuals across a set of opinion statements [9]. In 1993, Stephenson further formalized the philosophical foundation of the method—operant subjectivity—and established the core concept of the concourse: the natural universe of self-referential statements and meanings surrounding a given topic. By sampling representative statements from the concourse and administering Q sorting procedures, researchers can identify distinct, shared subjective perspectives within a population, bridging qualitative depth with quantitative rigor [10]. For lymphoma patients with highly individualized illness experiences, this method enables systematic identification of patterned experiential perspectives, providing a more nuanced evidence base for person-centered care.

Experience-Based Co-Design (EBCD) is a participatory quality improvement framework widely applied in healthcare settings, which centers the lived experiences of both service users and healthcare providers [11]. The EBCD approach guided the full design process of this study. Specifically, during concourse development, we conducted semi-structured interviews from the dual perspectives of patients and clinical staff to collect typical illness experience narratives and identify clinically relevant care priorities. This participatory strategy ensures that the Q sample comprehensively reflects patients’ real-world experiences while aligning with clinical practice contexts.

Patient-reported outcomes (PROs) refer to direct reports from patients regarding their health status, health behaviors, or care experiences, typically collected via standardized questionnaires [12]. Building on a 2025 conceptual framework proposed by Zhang et al., which categorizes mainstream PROs into six distinct domains—health-related quality of life, health preferences, health behaviors, symptom burden, functional status, and patient-reported experiences—this study performed systematic refinement of the Q sample based on this six-dimensional framework to ensure comprehensive coverage of core experiential dimensions. In turn, the distinct subjective perspectives identified via Q methodology will provide empirical evidence for the future development of lymphoma-specific patient-reported outcome measures (PROMs), supporting optimized item design and improved clinical sensitivity of assessment tools.

The purpose of this study is to identify distinct subjective perspectives of illness experiences among lymphoma patients receiving inpatient cyclic treatment using Q methodology, characterize the core features and corresponding population profiles of each perspective, enrich the patient journey map for the active treatment phase, and lay an empirical foundation for optimizing personalized clinical care and developing lymphoma-specific patient-reported outcome measures.

## 2. Materials and Methods

We used the work of William Stephenson on operant subjectivity to guide this research. Under this theoretical framework, we employed a standardized Q-methodology design integrating qualitative and quantitative approaches to systematically characterize the subjective illness perspectives of lymphoma patients undergoing active treatment. The study was implemented in six sequential stages: (1) concourse development via semi-structured interviews and literature review; (2) Q sample refinement through expert consultation and conceptual framework validation; (3) purposive sampling of participants (P sample); (4) face-to-face Q sorting administration; (5) factor extraction and rotation using PQMethod software (v2.35) [13]; and (6) qualitative interpretation and profiling of the identified factors.

This study is reported in accordance with the Q-methodology research reporting checklist [14], and the qualitative components comply with the Consolidated Criteria for Reporting Qualitative Studies (COREQ) checklist [15].

### 2.1. Study Design

#### 2.1.1. Construct the Concourse

The first step in Q-methodological research design is to collect diverse perspectives on the research topic through multiple channels to form a comprehensive statement pool, namely the concourse. In this study, the concourse was developed from three sources:

First, we conducted a systematic retrieval of lymphoma-specific patient-reported outcome measures, including the FACT-Lym, EORTC QLQ-NHL, EORTC QLQ-HL, and QLICP-LY [16,17,18,19], and extracted core items related to illness experience from these scales. Second, we performed a meta-synthesis of qualitative studies on lymphoma treatment experiences to supplement experiential statements from existing research evidence. Third, we conducted semi-structured interviews with two stakeholder groups to verify clinical applicability and complement real-world experiential details:

Patient interviews: Two inpatients with lymphoma were recruited. Interviews were guided by a semi-structured outline covering four dimensions: physical symptoms, psychological state, social life, and treatment experience. Restricted by the ward environment and the first-contact relationship with participants, interviews were documented via on-site written notes without audio recording to reduce patient discomfort. All interviews were conducted by two research team members trained in qualitative research methods. Healthcare professional interviews: Eighteen clinical healthcare providers (4 physicians and 14 nurses) were enrolled. Interviews lasted 16 to 72 min. All interviews were audio-recorded with written informed consent and transcribed verbatim afterwards. Thematic analysis was applied to extract core clinical concerns and typical patient experience statements. Interviews were administered by trained members of the research team.

Ultimately, the initial concourse was formed, containing 59 statements covering four dimensions of illness experience: physical, psychological, social, and treatment-related.

#### 2.1.2. Determine the Q-Set

The Q-set comprises representative statements from the concourse, employed for Q-sorting among research subjects. Adhering to principles of comprehensiveness, heterogeneity, and representativeness, a standardized expert consultation screening procedure was adopted:

First, a multidisciplinary expert panel of 11 members (covering oncology clinical medicine, nursing management, nursing humanities, and psychological nursing) was convened. All 59 initial statements from the concourse were scored by experts on a 5-point Likert scale in terms of their relevance to lymphoma patients’ illness experience and representativeness of real clinical scenarios. Second, the coefficient of variation (CV) of each statement was calculated. Statements with a CV ≥ 0.25, which indicated insufficient expert consensus on the item’s importance, were excluded. Finally, the research team refined and polished the wording of retained statements to ensure clear, concise, and patient-friendly expression, and unified the statement format. After final verification, a total of 39 statements were retained to form the final Q-set. The detailed screening information of all 59 initial concourse statements is shown in Table 1.

### 2.2. Data Collection

#### 2.2.1. Select Participants

The participant group that ranked the Q-set is termed the P-set. This study was conducted in the Hematology Department of a tertiary hospital in Nanjing. Between June and August 2025, a convenience sampling method combined with maximum difference sampling was employed to select a small sample of hospitalized lymphoma patients as research subjects. The sample size should constitute between one-half and one-third of the Q-sample size, not exceeding the Q-sample size. Inclusion criteria: patients diagnosed with lymphoma according to the diagnosis and treatment guidelines and confirmed by pathological examination; age ≥ 18 years and ≤80 years; hospitalized for treatment; normal cognition with intact verbal expression abilities; educational attainment of primary school level or above, capable of independently completing questionnaires; aware of their medical condition. Exclusion criteria: presence of psychiatric disorders or cognitive impairment; concurrent malignant tumors or significant organ dysfunction (cardiac, hepatic, renal, etc.). This study was conducted in accordance with the Declaration of Helsinki, and ethical approval was obtained from the Ethics Committee of the First Affiliated Hospital of Nanjing Medical University (Jiangsu Province Hospital). Written informed consent was acquired from all participants prior to enrollment.

#### 2.2.2. Perform Q-Sorting

**Figure 1:**
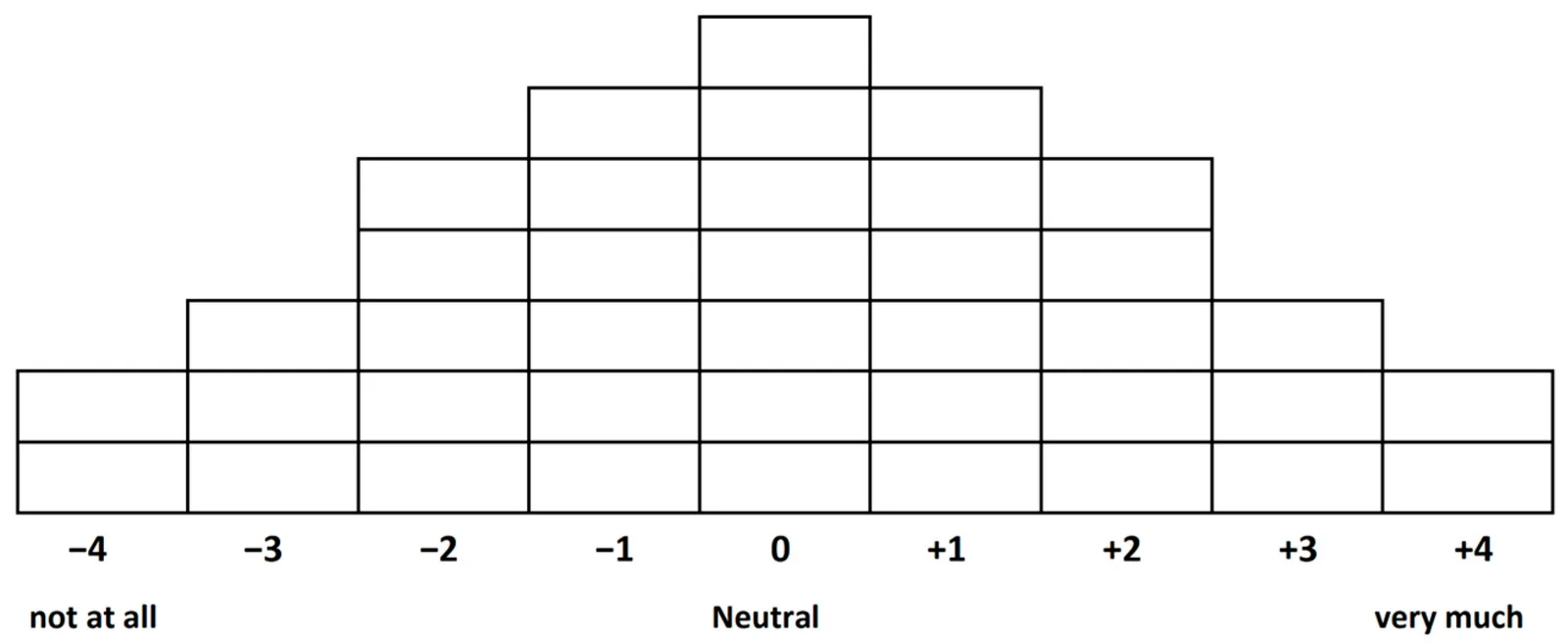
Table of Near-Normal Distributions at Level 9.

A predefined nine-point quasi-normal distribution table was employed, with a sorting scale ranging from +4 to −4. The anchoring term used was “conformity”, ranging from “very much in line with my experience” to “not at all in line with my experience”, as detailed in Figure 1. A face-to-face Q-sorting method was adopted. The researcher distributed 39 Q-statement cards and one normal distribution table to the research subjects, providing detailed instructions on the procedure: (1) Read all Q-statements to form a basic impression, then divide cards into three piles: ‘Relevant’, ‘Neutral’, ‘Irrelevant’; (2) place cards leaning towards ‘Relevant’ sequentially from the right, cards leaning towards ‘Irrelevant’ sequentially from the left, and finally position cards leaning towards ‘Neutral’ in the remaining central space; (3) the degree of fit in the table increases horizontally from left to right; within each column, there is no hierarchy of fit, and adjustments may be made dynamically during sorting; (4) justifications may be provided for Q cards placed at the extreme points (+4, −4).

### 2.3. Data Analysis

Enter the file of statements and Q-sorting results into the PQMethod software. Factor extraction employed Principal Component Analysis (PCA), statistically the optimal factor solution method for examining multiple variable correlations and revealing internal structures. Factor rotation utilized Varimax Rotation (VR), maximizing variance between factors to ensure subjects exhibit high factor loadings only on single factors. Retained factors must satisfy the following criteria: (1) According to Kaiser’s criterion, factor eigenvalues must exceed 1, and at least two Q-sortings must be assigned to each factor; (2) the absolute value of factor loadings must exceed 2.58 × (1 ÷ √number of Q-sample cases), with no cross-factor Q-sort mappings; (3) factors must exhibit sufficient variance explanation, with cumulative variance explained by all factors exceeding 40%.

### 2.4. Presentation of Findings

Interpreting the output of PQMethod software is best achieved by combining factor outputs, standardized factor scores (Z-scores), and distinguishing statements. Explanations for subject selection criteria may also aid factor interpretation. Z-scores measure the significance of each statement’s association with its assigned factor, while distinguishing statements identify those that make a factor’s perspective statistically significant (e.g., *p* < 0.01) relative to others.

## 3. Results

Data were collected from June to August 2025. The P-sample comprised 17 hospitalized lymphoma patients aged between 25 and 73 years (M = 51.94, SD = 17.28), including 9 males and 8 females. Diffuse large B-cell lymphoma was the predominant subtype (35.3%). Other detailed demographic characteristics are presented in Table 2.

The PQMethod software automatically extracted eight factors. Following systematic comparison of alternative factor structures, a four-factor solution was retained, cumulatively explaining 61% of the variance. During factor extraction, the significance threshold for factor loadings was adjusted from 0.41 to 0.45 (approximately 2.81 × SE, exceeding the standard *p* < 0.01 criterion of 2.58 × SE) to minimize confounding loadings and ensure that each retained factor was defined by sorters clearly belonging to that perspective. In the final four-factor solution, two participants showed confounded loadings (2/17 = 11.76%, P2, P3) and one participant showed no significant loading on any factor (1/17 = 5.88%, P17), with the remaining participants each loading uniquely on a single factor, effectively preserving the perspectives of the majority of participants.

A correlation analysis was conducted on the four factors, and the results showed that the Pearson correlation coefficients between the factors ranged from 0.316 to 0.508, all indicating moderate positive correlations. Factor 1 showed the highest correlation with Factor 3 (r = 0.508), suggesting that the two share common experiences in terms of the burden of physical symptoms, but differ fundamentally in their core psychological orientation and stage of disease progression; Factor 4 had correlation coefficients below 0.35 with all other factors, representing the most independent subjective perspective. Overall, the four factors share common experiential characteristics within the same patient population whilst each possessing a distinct and clearly identifiable core perspective; there are no redundant factors with significant overlap, and the factor structure demonstrates good validity.

The five Q-statements with higher Z-scores were designated as ‘strongly agree’ statements for this factor, while the five Q-statements with lower Z-scores were designated as ‘strongly disagree’ statements. These, together with the distinguishing statements, were compiled into a factor interpretation checklist, as detailed in Table 3.

### 3.1. Factor 1: Need for a Healing Environment Amid Severe Adverse Reactions

Patients sharing this perspective (P4, P7, P8, P14) focus their attention on day-to-day treatment realities, with physical symptoms and the inpatient environment jointly forming the core of their illness experience. They experience pronounced treatment-related adverse reactions, and fatigue, gastrointestinal discomfort and sleep disturbance dominate their daily feelings. Meanwhile, outdated ward facilities, crowded noisy surroundings and negative emotions from roommates further amplify their distress.

P4 (71-year-old female, diffuse large B-cell lymphoma), who has a hemiplegic spouse requiring care and is burdened with household chores at home, finds hospitalization “somewhat of a respite” and even sleeps better in hospital than at home, though the financial pressure of treatment costs relative to her pension remains a constant concern. P7 (37-year-old male, NK/T-cell lymphoma) explicitly states that he avoids searching for disease-related information online, deliberately steers clear of unfavorable treatment stories shared by older patients, and proposes that “patients with similar conditions and positive attitudes be placed in the same ward” to protect his emotional state through environmental selection. For them, illness experience is not an abstract judgment of prognosis, but the physical discomfort and daily ward life they face firsthand. They mainly adopt avoidance and buffering as coping strategies: they do not dwell on long-term outcomes, but focus on getting through each cycle of treatment reactions.

### 3.2. Factor 2: Active Emotional Adjustment and Adaptation to Treatment Rhythm

This group of patients (P1, P6, P9, P15, P16) consistently reflect that while acknowledging the constraints imposed by illness, they choose to cope proactively, actively manage their health and make full use of available support resources. Their shared trait is that despite physical discomforts such as fatigue, alopecia and taste alterations, they are not emotionally overwhelmed by the disease.

P1 (25-year-old female, Hodgkin lymphoma) actively cooperates with name and bed number verification at each infusion change. P6 (61-year-old female, diffuse large B-cell lymphoma) wears a wig when walking in the park and feels “just like a normal person”. P15 (42-year-old male, diffuse large B-cell lymphoma) remains in a lighthearted mood during monoclonal antibody maintenance therapy, noting that “there’s not too much pressure, I just focus on making a living”. P16 (29-year-old female, Hodgkin lymphoma) has strong comprehension and actively explains her spiritual sustenance rooted in Buddhist and Taoist thoughts.

They do not evade the reality of illness: they are fully aware of their physical changes, but choose to respond with concrete actions, such as taking the initiative to inquire about CAR-T therapy and transplantation, maintaining personal hobbies (growing flowers, watching football matches) and preserving family and social roles. Patient P6 feels distressed that her son travels back and forth for her treatment but does not refuse his support; P9 (45-year-old female, follicular lymphoma) persists in treatment accompanied by her family despite her poor complexion. They hold an attitude of “facing and accepting” the disease: they are neither blindly optimistic nor trapped in excessive panic; while recognizing the severity of the disease, they are able to focus on current treatment and daily life.

### 3.3. Factor 3: Fear of Disease Recurrence Amid Chronic Symptom Distress

This factor comprises 3 patients: P5 (73-year-old male, follicular lymphoma), P10 (57-year-old female, small B-cell lymphoma) and P13 (70-year-old male, angioimmunoblastic T-cell lymphoma). They are all elderly patients, mostly with relapsed or advanced-stage disease, generally with lower educational levels, mostly retired, and with relatively limited social support. Most patients with this perspective have experienced a long disease course and repeated treatment lines. Acute chemotherapy toxicities have subsided, but chronic consumptive symptoms such as fatigue, bone and joint pain, and weight loss persist. Their core distress no longer stems from immediate treatment reactions, but from deep-seated concerns about disease progression and recurrence, as well as a sense of loss of control over the future.

These patients tend to be introverted and reluctant to reveal their emotions; they mostly internalize their worries, or divert their attention through religion or daily hobbies. Patient P5 initially mistook his condition for pancreatic cancer at diagnosis and has a deep awareness of the severity of the disease, believing that communication with other patients is of little value. Patient P10 gains spiritual support through Christianity, and silently bears the economic pressure and physical discomfort, rarely taking the initiative to express her distress. Patient P13 is taciturn, rarely shares his feelings actively, and hides his worries about the disease behind his usual silence.

### 3.4. Factor 4: Resilient Persistence Under Heavy Financial Pressure

Patients with this perspective retain relatively intact physical function and are not completely overwhelmed by acute treatment reactions; instead, the financial burden of illness and its long-term impact on family life constitute their core source of stress. This factor includes 2 patients: P11 (44-year-old male, diffuse large B-cell lymphoma) and P12 (35-year-old male, NK/T-cell lymphoma). They are both young and middle-aged employed males who serve as the main economic pillar of their families. They are more concerned about the impact of treatment costs, income interruption and subsequent rehabilitation on family finances and life plans, and this realistic pressure even outweighs the distress caused by physical symptoms.

They are generally reserved in character, do not show emotions outwardly, are polite and maintain clear personal boundaries, and rarely take the initiative to talk about their own pain and concerns. P11 is hospitalized alone, polite but quiet, with “plenty of unspoken emotions inside” that he chooses not to voice. P12 has had a near-death experience; after going through this life-and-death trial, he is well aware of the economic cost of subsequent treatment but rarely mentions it on his own initiative.

### 3.5. Summary of Perspectives and Consensus Statements

The four subjective perspectives presented above comprehensively depict the differentiated pathways of illness experience among lymphoma patients during inpatient treatment. The spectrum ranges from being overwhelmed by acute physical distress in the initial treatment phase, to gradual adaptation to the cyclic treatment rhythm, then to persistent anxiety about disease recurrence in the chronic disease stage, and finally to the unique financial burden predicament faced by young and middle-aged patients. These findings cover multiple dimensions and disease stages of patients’ lived experience, and further validate the unique value of Q methodology in uncovering the structure of subjectivity: even under the same disease context, patients’ meaning-making of illness is not homogeneous, but presents distinct group differentiation shaped by individual circumstances.

Despite the divergent emphases of the four perspectives, all patients reached cross-group consensus on several core dimensions of illness experience. Strong consensus, defined as statements with no statistically significant difference across all factors at the 0.01 level (*p* > 0.01), was found for two items: all patients endured complex emotional distress including anxiety, depression and guilt (S17), and generally reported diminished independence and increased reliance on others for daily assistance (S29). Moderate consensus (no significant difference across all factors at the 0.05 level, *p* > 0.05) was identified for two universal experiences shared across all perspectives: the sense of losing control over daily life and facing an uncertain future (S20), and the fundamental shift of life priorities towards health and family companionship (S23).

## 4. Discussion

The physical environment of hospitals is closely linked to patients’ emotional well-being and health-related quality of life, and is widely recognized as a core element shaping the inpatient treatment experience. The material attributes of the hospital environment exert effects through three pathways: environmental features delivering sensory comfort and security assurance (e.g., clean sanitary conditions and clear wayfinding signage), shared spaces that balance social interaction and privacy protection, and professional support embedded in therapeutic practices that reflects care quality [20].

Patients in Factor 1, who suffered from pronounced treatment-related adverse reactions and were physically and mentally exhausted, expressed low satisfaction and limited perceived control over environmental features and shared ward spaces. From the theoretical perspective of embodied cognition, the body, cognition and environment form a dynamic unity: patients’ cognitive processing of illness is situated within the clinical setting and evolves along with disease progression [21].Consistent with Ulrich’s supportive design theory, hospital environments that foster perceptions of control, social support and positive distraction can effectively mitigate stress responses in patients undergoing intensive treatment [22].

Targeted optimization focusing on soundscapes, lighting, greenery, privacy protection and humanistic details is recommended to cultivate a healing environment conducive to physical recovery and emotional well-being. Specifically, biophilic interventions—including indoor plants, green landscapes, natural materials, optimized daylight access, healing gardens and nature-themed digital media—have been evidenced to enhance stress recovery, reduce pain perception, improve emotional health and sleep quality, and boost nursing satisfaction [23]. For wards constrained by physical conditions, virtual reality technology can serve as an alternative medium to deliver simulated healing environments, thereby supporting stress management and symptom control for patients in the acute reaction phase.

Factor 2 accounts for the largest proportion of patients in this study, representing the most representative psychological adaptation pattern of lymphoma patients after entering the stable treatment phase. Despite enduring treatment-related physical discomforts such as fatigue, alopecia and taste alteration, these patients do not sink into a state of passive tolerance. Instead, on the premise of accepting the reality of illness, they proactively adjust their life rhythm and mobilize internal and external resources to cope with the disease. This positive psychological transformation aligns closely with the core connotation of post-traumatic growth (PTG). As a major traumatic event, cancer not only exerts negative emotional impact, but may also trigger profound positive psychological changes, including re-examination of life, deepened interpersonal relationships, discovery of personal strengths and perception of new life possibilities [24].

From the perspective of medical coping modes, the behavioral profile of this group conforms to the “confrontation” strategy in Feifel’s three-factor model: they actively seek diagnosis and treatment information, participate in treatment decisions, and maintain daily interests and social roles, confronting the impact of illness with a proactive problem-solving stance. This stands in sharp contrast to the avoidant coping style of Factor 1 and the introverted passive state of Factor 3. Existing hematological oncology research has confirmed that the development of PTG is not a simple emotional outcome, but follows a sequential mediation pathway of “social support → coping strategies (avoidance/acceptance-resignation) → depression → PTG”, among which active confrontation coping serves as a key bridge linking social support to positive psychological growth [25]. In this study, patients in Factor 2 generally perceive and fully leverage family support, adopt active coping to buffer negative emotions, and subsequently reconstruct life priorities and rethink life meaning, which verifies the applicability of this mechanism among lymphoma patients.

In clinical practice, care for this patient group should not be confined to physical symptom management, but should also focus on preserving and fostering their potential for post-traumatic growth. By strengthening patients’ family and social support systems, empowering their autonomy in disease self-management, and guiding them to explore life value after illness, the positive effect of active coping can be further consolidated, and patients’ long-term psychological resilience and health-related quality of life can be improved.

Death anxiety (DA) constitutes a core attribute of existential suffering, denoting an emotional state of unease, apprehension, and dread arising from an individual’s awareness of death’s inevitability. Common coping mechanisms involve experiential avoidance through various forms of distraction. This aligns with the cross-factor consensus identified in this study: worry about disease worsening or recurrence, and the implicit association between disease progression and death, is a universal experience shared by patients across all four perspectives. Specifically, patients in Factor 3, mostly elderly individuals with relapsed or advanced-stage disease, exhibit the most prominent death anxiety. Their introverted traits and tendency to conceal worries make this form of existential distress highly hidden in clinical practice.

Grounded in stress and coping theory, experiential avoidance (EA) constitutes a psychological response linked to fear of disease progression and negative emotions, encompassing cognitive, emotional, and behavioral avoidance. While potentially beneficial as a short-term coping strategy, its prolonged use intensifies and increases the frequency of distress [26]. A cross-sectional study of Chinese cancer patients found that EA is a predictor of DA, while a sense of life meaning exhibits a moderate negative correlation with DA and mediates this relationship [27]. Lymphoma patients’ death anxiety can be addressed through meaning therapy and hope interventions, encouraging individuals to explore life meaning by leveraging character strengths. For highly concealed patient groups such as Factor 3, routine screening for recurrence anxiety and death distress should be incorporated into daily nursing practice. Furthermore, patients experiencing death anxiety seek support within intimate relationships; integrating meaning therapy with couples therapy can fulfill patients’ attachment needs to loved ones, benefiting both parties.

Financial toxicity (FT) impacts patients and their families through both objective financial hardship and subjective financial distress. The former manifests as medication-related out-of-pocket expenses and transport costs, while the latter encompasses material, psychological, and behavioral dimensions: patients experience reduced income due to treatment-related work interruptions, develop negative emotions such as anxiety and depression, and subsequently adapt by cutting daily expenditures or altering established lifestyles [28]. A cross-sectional study on FT among Chinese non-Hodgkin lymphoma patients revealed that 39.98% exhibited severe FT, with unemployment, lower household income, and increased chemotherapy cycles identified as contributing factors [29].

Patients in Factor 4 align closely with this high-risk demographic profile. As young and middle-aged employed males and the primary economic pillar of their families, they bear the dual pressure of direct medical costs and interrupted household income, and this realistic burden often outweighs the distress caused by physical symptoms. Notably, influenced by social role norms, these patients tend to internalize financial pressure and rarely take the initiative to express economic concerns, making this need highly overlooked in routine clinical care. Long-term FT management may compromise treatment adherence or lead to unhealthy medical decision-making. Based on the Information–Motivation–Behavioral Skills model, healthcare professionals can employ networked cognitive behavioral therapy and motivational interviewing to activate lymphoma patients’ intrinsic rehabilitation motivation, thereby enhancing treatment adherence and mitigating long-term impacts. Regarding external support, clinicians may provide informational assistance and encourage patients to engage in discussions about costs and available healthcare resources, rather than waiting for patients to raise concerns. Additionally, beyond financial support, the state should implement skills training programs for caregivers.

Beyond the specific clinical implications of each perspective, the present study underscores the broader value of Q-methodology as a tool for informing patient-centered care. Unlike standardized questionnaires that aggregate responses into mean scores, Q-methodology preserves the internal coherence of each individual’s viewpoint, enabling researchers and clinicians to recognize patterns of shared subjectivity that might otherwise remain invisible. As Dennis argued, Q-methodology is particularly suited to nursing and healthcare research precisely because it places the subjective experience of the patient at the center of inquiry [30]. In the context of lymphoma care, where patients with similar diagnoses and treatment regimens may nevertheless experience profoundly different illness trajectories, the ability to systematically identify distinct experiential perspectives provides an evidence base for differentiated supportive care strategies. The distinguishing statements identified for each factor in this study could, in future work, serve as the foundation for brief screening tools that help clinicians quickly recognize which perspective a newly admitted patient may hold—and tailor their communication and support accordingly.

## 5. Conclusions

Patient experience data provide direct insights into how individuals perceive the impact of their illness on daily functioning and quality of life. Under the “patient-centered” philosophy, it is essential to give full consideration to patients’ voices. Q-methodology, combining quantitative and qualitative techniques, employs data-driven clustering instead of predefined structures to explore the subjectivity of small-sample research subjects, and holds great potential as an interpretative approach in nursing research.

This study identified complex and diverse experiences among hospitalized lymphoma patients, summarizing four distinct subjective perspectives. Differentiated care strategies should be adopted for patients with different factor characteristics, such as optimizing the inpatient healing environment, fostering active coping and post-traumatic growth, alleviating recurrence anxiety and chronic distress, and mitigating financial toxicity. These findings provide empirical evidence for the development of clinical personalized supportive care and lymphoma-specific patient-reported outcome measures.

## Figures and Tables

**Table 1:** Detailed screening results of initial concourse statements.

Course	CV	Expert Consensus	Q-Set
1. I can feel swollen lymph nodes (in the neck, armpits, groin, etc.)	0.08		S5
2. I have experienced fever/chills	0.13		S7
3. I am prone to night sweats and excessive perspiration	0.00		S2
4. I have pain/discomfort in my joints, muscles, or bones	0.23		S6
5. I notice signs of anaemia such as pallor and pale lips	0.24		S8
6. I have cough and sputum	0.28		×
7. I experience breathlessness, chest tightness, or palpitations	0.23		S15
8. My heart beats very fast	0.28	Merged into Item 7	×
9. I experience sleep disturbances (difficulty falling asleep, vivid dreams, early waking, etc.)	0.18		S27
10. I have throat symptoms (hoarseness, sensation of a lump in the throat, sore throat, etc.)	0.23		S11
11. I have mouth ulcers, dry mouth, or a bitter taste	0.15		S10
12. My sense of taste has changed	0.21	Merged into Item 14	×
13. I have a poor appetite	0.18	Merged into Item 14	×
14. I experience gastrointestinal issues (nausea, vomiting, constipation, bloating, diarrhoea, loss of appetite, etc.)	0.15		S9
15. Bloating and burping	0.26		×
16. I have lost weight/feel weak/appear emaciated	0.14		S4
17. I suffer from constipation.	0.21	Merged into Item 14	×
18. I suffer from diarrhea.	0.21	Merged into Item 14	×
19. I have numbness/tingling in hands and feet	0.18		S12
20. I experience dizziness/headaches	0.24		S13
21. I bruise and bleed easily.	0.30		×
22. My interest in sex has decreased.	0.34		×
23. My skin exhibits itching, bleeding, and cracking	0.11		S3
24. My physical appearance has changed (hair loss, facial deformity, limb swelling, etc.)	0.19		S16
25. I experience fatigue and poor stamina	0.15		S1
26. I have cognitive issues (impaired memory, difficulty concentrating, slowed reactions, etc.)	0.18		S14
27. I need help with self-care (using the toilet, walking, eating, washing)	0.24	My independence diminishes, leading to reliance on others and need for assistance	S29
28. I need help with daily tasks (housework, shopping, cycling)	0.18		
29. My overall health condition is good	0.32		×
30. I feel complex emotions such as anxiety, depression, or guilt	0.18		S17
31. I am prone to anger/nervousness/outbursts	0.06		S18
32. I worry about the disease worsening/relapsing, associating it with death	0.06		S21
33. I develop avoidance behaviour, refusing to think about the illness or treatment to block out the pain	0.13		S19
34. I am easily affected by others’ opinions, experiencing diminished self-esteem and stigmatisation	0.15		S22
35. I feel my life has spun out of control, with an uncertain future ahead	0.18		S20
36. I maintain hope for recovery, aspiring to return to normal life	0.14		S26
37. I have undergone post-traumatic growth, gradually becoming accustomed to/adapting to/accepting the illness and treatment	0.21		S24
38. I seek support/spiritual solace through religion/faith	0.23		S25
39. My outlook on life/priorities have shifted, placing greater value on health and family companionship	0.23		S23
40. Everything will be fine	0.35		×
41. I find self-worth through fulfilling some family responsibilities	0.00		S33
42. I feel my personal meaning/identity is shattered, with loss of social functioning	0.13		S28
43. I find myself socially isolated (reduced social activities/lack of social acceptance)	0.06		S30
44. I am a burden and cannot meet my family’s needs	0.08	Contradicts Item 41	×
45. My family relationships are strained/intimate relationships are disrupted	0.21		S32
46. I currently feel it is meaningless to consider returning to work/school/society	0.27		×
47. My daily activities/leisure pursuits/hobbies are restricted	0.08		S34
48. I have come to recognize who my true friends are	0.30		×
49. I receive support from family/medical staff/fellow patients	0.21		S35
50. I bear heavy financial burdens/significant economic pressure	0.00		S31
51. There are ways to obtain additional help outside the hospital (social workers, support groups, supplementary health insurance, etc.)	0.13	Duplicated with Item 49	×
52. The hospital environment and medical facilities meet my treatment needs	0.21		S38
53. The transportation, consultation, and treatment processes are convenient	0.30		×
54. I know how to communicate with healthcare staff about my health problems	0.38		×
55. I take the initiative to report abnormal symptoms or signs to healthcare staff and ask how to take care of myself	0.26		×
56. The information I receive (possible side effects, home management, returning to society, etc.) is sufficient	0.00	I can access guidance on my condition and treatment, enabling effective self-management (adherence to prescribed medication, diet, exercise)	S36
57. I actively take charge of my own health management (following medical advice on diet, exercise, medication, treatment, and follow-up visits)	0.00		
58. I have limited contact with my treatment team, finding it difficult to gain the attention and consideration of healthcare professionals	0.20		S37
59. I am satisfied with the current treatment outcomes	0.06		S39

**Table 2:** Participant demographics.

Variable	*n*	%
**Sex**		
Male	9	52.94%
Female	8	47.06%
**Age (years)**		
20~30	2	11.76%
31~40	3	17.65%
41~50	3	17.65%
51~60	1	5.88%
>60	8	47.06%
**Marital status**		
Unmarried	15	88.24%
Married	2	11.76%
**Occupation**		
Retired	8	47.06%
Corporate employee	7	41.18%
Technical personnel	2	11.76%
**Employment status** ^※^		
Resigned	4	23.53%
On leave	3	17.65%
Full-time	2	11.76%
**Level of education**		
Primary school and below	1	5.88%
Lower secondary school	4	23.53%
Vocational secondary school	1	5.88%
Upper secondary school	2	11.76%
College	3	17.65%
Undergraduate degree and above	6	35.30%
**Types of medical insurance reimbursement**		
Employee Basic Medical Insurance	14	82.36%
Residents Basic Medical Insurance	2	11.76%
Provincial Medical Insurance	1	5.88%
**Lymphoma Subtypes**		
Diffuse large B-cell lymphoma	6	35.30%
Natural killer/T-cell lymphoma	3	17.66%
Classic Hodgkin lymphoma	2	11.76%
Follicular lymphoma	2	11.76%
Small B-cell lymphoma	1	5.88%
Mantle cell lymphoma	1	5.88%
Angioimmunoblastic T-cell lymphoma	1	5.88%
Intestinal T-cell lymphoma	1	5.88%
**Disease stage**		
I	1	5.88%
II	2	11.76%
III	4	23.53%
IV	10	58.83%
**Treatment methods**		
Cyclical treatment (chemotherapy + targeted therapy)	9	52.94%
Monoclonal antibody only	2	11.76%
Re-treatment for recurrence	6	35.30%
**Duration since diagnosis**		
<6 months	9	52.94%
≥6 months, <1 year	3	17.66%
≥1 year, <2 years	2	11.76%
≥2 years, <5 years	2	11.76%
≥5 years	1	5.88%

Note: ^※^ Employment status includes only non-retired personnel, with a total of 9 individuals.

**Table 3:** Factor Interpretation Checklist.

Factor	Top 5	Bottom 5	Distinguishing Statements
Factor 1 (P4, P7, P8, P14): Need for a healing environment amid severe adverse reactions	S1, S9, S2, S27, S10	S25, S5, S11, S12, S22	S1: +4, 2.38 (*p* < 0.01) S27: +3, 1.09 (*p* < 0.01), S10: +3, 0.97 (*p* < 0.05) S15: +2, 0.83 (*p* < 0.01) S8: +1, 0.33 (*p* < 0.05) S30: 0, 0.14 (*p* < 0.01), S18: 0, 0.14 (*p* < 0.01) S38: −2, −1.03 (*p* < 0.01) S25: −4, −2.12 (*p* < 0.01)
Factor 2 (P1, P6, P9, P15, P16): Active emotional adjustment and adaptation to treatment rhythm	S26, S2, S3, S34, S36	S19, S28, S37, S32, S18	S34: +3, 1.28 (*p* < 0.01) S6: 0, −0.11 (*p* < 0.01) S4: −1, −0.61 (*p* < 0.01) S25: −2, −1.26 (*p* < 0.01) S28: −4, −1.52 (*p* < 0.05), S19: −4, −2.05 (*p* < 0.01)
Factor 3 (P5, P10, P13): Fear of disease recurrence amid chronic symptom distress	S2, S9, S1, S6, S4	S7, S3, S11, S12, S14	S21: +2, 1.29 (*p* < 0.05) S10: −2, −0.90 (*p* < 0.05) S3: −4, −1.72 (*p* < 0.01), S7: −4, −2.10 (*p* < 0.01)
Factor 4 (P11, P12): Resilient persistence under heavy financial pressure	S24, S31, S26, S1, S8	S34, S30, S5, S14, S18	S31: +4, 1.82 (*p* < 0.01) S8: +3, 1.18 (*p* < 0.05) S11: 0, 0.00 (*p* < 0.01), S22: 0, −0.04 (*p* < 0.05) S2: −1, −0.67 (*p* < 0.01) S27: −2, −1.03 (*p* < 0.01) S34: −4, −1.82 (*p* < 0.01)

## Data Availability

The data presented in this study are available upon request from the corresponding author.
